# Sex Differences in Excess Mortality Among Waitlisted Kidney, Heart, and Liver Transplant Candidates

**DOI:** 10.1097/TXD.0000000000001856

**Published:** 2025-08-22

**Authors:** Amanda Jean Vinson, Xun Zhang, Lauren T. Grinspan, Bethany J. Foster

**Affiliations:** 1 Nephrology Division, Department of Medicine, Dalhousie University, Halifax, NS, Canada.; 2 Research Institute of the McGill University Health Centre, Montréal, QC, Canada.; 3 Recanati/Miller Transplantation Institute, Icahn School of Medicine at Mount Sinai, New York, NY.; 4 Division of Nephrology, Department of Pediatrics, McGill University Faculty of Medicine and Health Sciences, Montreal, QC, Canada.; 5 Department of Epidemiology, Biostatistics, and Occupational Health, McGill University, Montreal, QC, Canada.

## Abstract

**Background.:**

Sex differences in excess mortality risk (ie, above expected in the age-, sex-, and race-matched general population) among candidates waitlisted for transplant may reflect sex bias in access to the waitlist, disparities in allocation policies, and/or sex differences in care for patients with organ failure.

**Methods.:**

We used time-dependent relative survival models to determine the relative excess risk of mortality in females compared with males recorded in the Scientific Registry of Transplant Recipients who were waitlisted for kidney, heart, or liver transplant from 1988 to 2019, accounting for the modifying effects of candidate age and listing era.

**Results.:**

Among 644 262 kidney and 106 353 heart candidates, excess mortality was higher in female than male kidney candidates <60 y, but lower in female kidney candidates ≥60 y and heart candidates ≥12 y; patterns did not differ by era. Among 259 230 liver candidates, patterns differed by era of waitlisting. Excess mortality was lower for female than male liver candidates 0–12 and 25–44 y, and higher for females than males 13–24 y, without differences by era. Excess mortality was lower for female than male liver candidates 45–59 y waitlisted 1988–2011, but not different by sex for those waitlisted 2012–2019. Among liver candidates ≥60 y, excess mortality did not differ by sex for those waitlisted 1988–2011 but was higher for females than males waitlisted 2012–2019.

**Conclusions.:**

The patterns of sex differences in excess mortality observed among waitlisted transplant candidates likely reflect the selection of healthier, lower-risk females than males for waitlisting and higher mortality risks for females with organ failure.

At all ages, males have higher mortality rates than females.^1^ Both biologic and social/environmental factors are believed to contribute to this difference.^2-5^ Given the male mortality bias in the general population,^1^ comparison of absolute mortality rates by sex among people with health conditions is problematic; to meaningfully compare sex differences in mortality requires examination of excess mortality rates (defined as the risk above that expected in the sex- and age-matched general population). Sex differences in excess mortality have been demonstrated after kidney^6,7^ and heart^8^ transplantation—to date, no studies have examined sex differences in excess mortality risk among patients with end-stage organ failure on the transplant waitlist. A study in Australia and New Zealand showed higher excess mortality in females than males treated with dialysis.^9^ However, this study examined all patients on dialysis, not just those active on the kidney transplant waitlist. Patients waitlisted for transplantation represent a selected subgroup of healthier patients without active contraindications to transplant, who are systematically different from those deemed ineligible or not referred for transplant. Therefore, while the results from Australia and New Zealand are meaningful, they cannot be extrapolated to the kidney transplant waitlist population. Females waitlisted for heart^10-13^ and liver^14^ transplant have been shown to have higher absolute mortality than males. Given the lower expected mortality rates in females than males, higher absolute mortality in females than males will translate into higher excess mortality. However, previous studies did not consider effect modification by candidate age or the possibility that sex differences in mortality may have changed over time. We aim to estimate differences in excess mortality between females and males of different ages waitlisted for kidney, heart, and liver transplantation in the United States. We also consider the possibility that sex differences in excess mortality may have changed over time.

## MATERIALS AND METHODS

### Data Sources and Population

This was a retrospective cohort study of patients recorded in the Scientific Registry of Transplant Recipients (SRTR) with a first registration date on the kidney, heart, or liver transplant waitlist between January 1, 1988, and December 31, 2019. We excluded patients listed for simultaneous multiorgan transplant and those on multiple organ type wait lists.

This study used data from the SRTR. The SRTR data system includes data on all donor, waitlisted candidates, and transplant recipients in the US, submitted by the members of the Organ Procurement and Transplantation Network. The Health Resources and Services Administration, U.S. Department of Health and Human Services provides oversight to the activities of the Organ Procurement and Transplantation Network and SRTR contractors. The data reported here have been supplied by the Hennepin Healthcare Research Institute as the contractor for the SRTR. The interpretation and reporting of these data are the responsibility of the author(s) and in no way should be seen as an official policy of or interpretation by the SRTR or the U.S. Government.

### Exposure and Outcome Definitions

#### Primary Exposure

The primary exposure was candidate sex. Previous studies demonstrated that recipient current age modifies the association between recipient sex and excess mortality after transplant^6,7^; it was anticipated that current age would similarly modify the association between candidate sex and excess mortality among waitlisted patients. Accordingly, we included a candidate current age by candidate sex interaction term in all models, allowing us to determine the association between candidate sex and excess mortality among waitlisted patients of different ages. Given that age is dynamic and biologic differences between males and females change with sexual development and senescence,^15,16^ current age was a time-varying variable categorized as 0–12 (pre-pubertal), 13–24 (adolescence and young adulthood), 25–44 (middle adulthood), 45–59 y (middle age), and ≥60 y (advanced age). These ages, representing developmental stages, were used in previous studies comparing graft outcomes by recipient sex post kidney, heart, and liver transplant.^6-8,17^

### Primary Outcome

The primary outcome was mortality, with observation censored at transplant, end of observation, or end of the study period (December 31, 2019, to avoid possible influence of the COVID-19 pandemic).

### Statistical Analysis

#### Association Between Candidate Sex and Excess Mortality

Time-dependent relative survival models^18,19^ with time-varying covariates were used to estimate the association between candidate sex and excess mortality, as previously described.^6-8^ We used relative survival models to estimate “relative excess risks” (RER), which are analogous to hazard ratios, and in this case represent the excess risk of mortality among female compared with male candidates on the transplant waitlist. Excess risk is defined as the risk among waitlisted patients above and beyond the expected risk in the age-, sex-, race-, and calendar year-matched general population.^19,20^ Time zero was the date of waitlist activation. Separate models were fitted for patients on the kidney, liver, and heart waitlists.

For each organ type, we created a dynamic dataset to determine the expected mortality risk for each patient over time, accounting for their changing age-related risk and the changing calendar year-related risk—age and calendar year of observation were updated as patients were followed over time.^20^ As previously described,^6,20^ each individual’s observation time was split into multiple 1-y intervals following waitlist activation. The expected probability of death in the general age- (based on the following categories: <1, 1–4, 5–9, and 5-y age intervals thereafter), race-, sex-, and calendar year-matched population was determined using the US National Vital Statistics^21^ general population mortality rates, and assuming a constant hazard within each interval.

The hazard function among waitlisted patients was modeled as the sum of the expected hazard and the excess hazard associated with organ failure/transplant waitlisting.^19^ To estimate the unconfounded, direct association between candidate sex and excess mortality for patients on each organ waitlist, models were adjusted for all available variables identified a priori to potentially act as confounders because of their known association with patient survival and their potential to be associated with candidate sex. Like in our previous analyses,^6-8^ we did not adjust for comorbidity burden given sex-specific general population mortality rates are similarly influenced by sex-related comorbidity risk and to adjust for these factors in the waitlisted but not the general population would introduce bias.

Because it is possible that the relation between candidate sex and excess mortality may have changed over time, we conducted additional analyses which included an interaction between sex and era (1988–1998, 1999–2008, 2009–2019). We then estimated the RER of mortality in female versus male candidates waitlisted for each organ type, stratified by era (1988–1998, 1999–2008, 2009–2019). For organs in which results differed by era, we also examined plots of mortality by year of waitlist activation to determine when the pattern changed.

### Crude Excess and Absolute Mortality Rates

We calculated crude excess ([observed − expected number of deaths within the same age interval] × 100/person-years within that age interval) and absolute (observed number of deaths in the age group × 100/person-years within that age group) mortality rates, with 95% confidence interval (CI), for males and females in each age group.^19^

Missing data were imputed using multiple imputation methods (15 imputations) based on the fully conditional specification method.^22^ Data analyses were performed using Statistical Analysis System 9.4 (SAS Institute, Carry, NC) and R version 4.0.3 (R Foundation for Statistical Computing, Vienna, Austria. http://www.R-project.org/). The study was approved by the McGill University Health Center Research Ethics Board.

## RESULTS

Over the study period, 644 262 patients were activated on the kidney transplant waitlist, 106 353 on the heart waitlist, and 259 230 on the liver waitlist; death occurred in 136 544 (21.2%) kidney, 23 179 (21.8%) heart, and 66 008 (25.5%) liver candidates. Patients could contribute observation time to multiple age intervals and calendar years. The median follow-up for kidney candidates was 2.4 y (interquartile range [IQR], 0.9–3.7), heart candidates 0.3 y (IQR, 0.1–1.9), and liver candidates 0.5 y (IQR, 0.1–2.0).

### Patient Characteristics

An overview of patient characteristics by organ type is provided in Tables 1 and 2. The majority of candidates waitlisted for each organ were male (60.8% of kidney, 73.9% of heart, and 61.6% of liver candidates) and most were White (63.0% of kidney, 78.9% of heart, and 85.6% of liver candidates). The most common cause of end-stage kidney disease (ESKD) was diabetes (32.8%), the most common cause of end-stage heart disease was myocarditis/cardiomyopathy (44.0%), and the leading cause of end-stage liver disease was hepatitis C (27.1%). The composition of the observed experience (ie, proportion of person-time) within each age interval for male and female waitlisted candidates for each organ is shown in Tables 3–5.

**TABLE 1.** Overview of patient characteristics (n, %) by organ type (unit of analysis here is the patient)

Variable	Kidney	Liver	Heart
Patients	644 262	259 230	106 353
Censoring			
Total death	136 544 (21.2)	66 008 (25.5)	23 179 (21.8)
Follow-up, y			
Median (IQR)	2.4 (0.9–3.7)	0.5 (0.1–2.0)	0.3 (0.1–1.9)
Male candidates, %	391 453 (60.8)	159 628 (61.6)	78 565 (73.9)
Age at listing, y			
Median (IQR)	52 (40–61)	53 (44–60)	52 (38–59)
Candidate race White Black Other	405 853 (63.0) 185 889 (28.9) 52 520 (8.2)	221 744 (85.6) 22 896 (8.8) 14 563 (5.6)	83 925 (78.9) 18 860 (17.7) 3568 (3.4)
Transplant listing year			
1988–1994 1995–1999 2000–2004 2005–2009 2010–2014 2015–2019	81 918 (12.7) 69 374 (10.8) 87 656 (13.6) 128 833 (20.0) 145 621 (22.6) 130 860 (20.3)	24 178 (9.3,) 61 387 (23.7) 43 107 (16.6) 46 757 (18.0) 51 084 (19.7) 56 868 (21.9)	22 118 (20.8) 17 739 (16.7) 14 282 (13.4) 14 404 (13.5) 17 178 (16.2) 20 632 (19.4)

IQR, interquartile range.

**TABLE 2.** Overview of patient characteristics (n, %) (unit of analysis here is the patient)

Kidney	
Primary disease	
Diabetes Hypertension Glomerulonephritis CAKUT Cystic Other Missing	211 347 (32.8) 137 182 (21.3) 120 486 (18.7) 13 621 (2.1) 47 761 (7.4) 101 333 (15.7) 12 532 (2.0)
On dialysis at list Yes No Missing	426 546 (66.2) 217 145 (33.7) 517 (0.1)
Liver	
Primary disease	
Congenital biliary atresia Alcohol-related liver disease Cancer Metabolic liver disease Fulminant liver failure Autoimmune conditions Hepatitis C Others Missing	8174 (3.2) 43 477 (16.8) 19 152 (7.4) 62 48 (2.4) 14 227 (5.5) 25 155 (9.7) 70 245 (27.1) 55 161 (21.3) 17 364 (6.7)
Heart	
Primary disease	
Congenital heart disease Coronary/ischemic disease Myocarditis/cardiomyopathy Other Missing	10 454 (9.8) 33 866 (31.8) 46 774 (44.0) 14 926 (14.0) 333 (0.3)

CAKUT, congenital anomalies of the kidneys and urinary tract.

**TABLE 3.** Composition of the contrasted experience of female and male kidney transplant candidates by candidate current age (unit of analysis is patient-years)

Variable	0–12 y		13–24 y		25–44 y		45–59 y		≥60 y	
	Females	Males	Females	Males	Females	Males	Females	Males	Females	Males
Person-years of observation	4698	7606	24 123	30 595	226 005	300 826	359 999	547 504	346 203	514 300
Candidate race, %										
White	74.5	71.0	59.7	62.7	51.5	55.2	53.6	56.1	55.9	61.9
Black	17.1	22.3	32.3	30.4	39.0	36.9	37.0	35.7	34.1	28.5
Other	8.5	6.7	8.0	6.9	9.4	7.9	9.4	8.3	10.0	9.6
Primary disease, %										
Diabetes	0.1	0.2	2.6	1.6	24.5	24.4	35.7	41.5	42.5	46.0
Hypertension	0.7	0.7	8.7	11.4	18.3	29.0	22.3	27.0	24.9	25.9
Glomerulonephritis	17.5	10.5	46.1	40.3	34.3	24.2	18.0	12.7	10.8	9.3
CAKUT	24.0	41.4	9.9	13.8	2.5	2.6	1.3	0.7	0.8	0.5
Cystic	9.0	5.3	2.9	2.2	4.1	4.1	9.2	6.8	7.1	4.8
Other	48.8	42.0	29.9	30.7	16.3	15.7	13.5	11.3	13.9	13.5
Missing	2.3	2.0	3.4	2.8	5.0	4.9	3.5	3.1	2.5	2.2
Listing year, %										
1988–1994	16.3	13.8	21.0	19.9	20.3	19.2	15.0	13.1	11.2	10.2
1995–1999	10.7	7.5	12.3	11.6	14.9	13.9	12.6	11.3	8.2	7.4
2000–2004	10.9	11.2	13.3	14.1	14.7	14.2	16.3	14.8	14.3	12.8
2005–2009	11.9	15.8	18.1	18.7	20.7	20.9	24.2	23.9	27.3	26.1
2010–2014	27.9	27.5	21.3	21.8	20.0	21.5	22.4	25.7	28.2	30.7
2015–2019	22.3	24.2	13.9	13.9	9.3	10.3	9.4	11.2	10.7	12.8
On dialysis at list										
Yes	62.2	56.7	64.3	62.4	67.1	69.9	72.7	76.7	76.7	79.4
No	37.8	43.3	35.7	37.6	32.9	30.1	27.3	23.3	23.3	20.6
Missing	3.0	1.9	1.1	0.8	0.0	0.0	0.0	0.0	0.0	0.0

Because the unit of analysis was person-time, rather than person, the characteristics presented are weighted by a factor derived from the number of person-years of observation and number of events. For example, 24% of the person-years contributed by female candidates between 0 and 12 y were by people with CAKUT as the primary disease.

CAKUT, congenital anomalies of the kidney and urinary tracts.

**TABLE 4.** Composition of the contrasted experience of female and male heart transplant candidates by candidate current age (unit of analysis is patient-years)

Variable	0–12 y		13–24 y		25–44 y		45–59 y		≥60 y	
	Females	Males	Females	Males	Females	Males	Females	Males	Females	Males
Person-years of observation	4698	7606	24 123	30 595	226 005	300 826	359 999	547 504	346 203	514 300
Candidate race, %										
White	74.3	79.7	73.5	77.7	68.2	72.3	71.0	81.7	78.0	87.7
Black	21.2	16.1	23.4	18.3	28.7	24.1	26.9	16.0	20.1	10.0
Other	4.5	4.2	3.1	3.9	3.1	3.6	2.2	2.3	1.8	2.3
Primary disease										
Congenital heart disease	45.3	56.4	32.7	38.1	13.7	11.2	5.8	2.9	3.4	2.2
Coronary/ischemic disease	1.3	1.0	2.2	1.6	7.9	12.1	21.2	40.6	28.6	54.2
Myocarditis/cardiomyopathy	39.2	27.2	44.6	39.0	63.0	60.1	59.4	42.3	53.8	31.8
Other	14.3	15.3	20.5	21.3	15.4	16.6	13.5	14.2	14.1	11.9
Missing	0.3	0.3	0.2	0.6	0.3	0.3	0.3	0.1	0.1	0.2
Listing year, %										
1988–1994	6.1	6.8	3.7	4.7	7.9	14.4	8.8	15.3	3.1	5.3
1995–1999	12.4	13.4	7.7	8.7	14.8	15.8	15.1	19.4	8.1	10.8
2000–2004	17.9	17.6	12.4	12.9	16.4	15.9	17.9	18.7	12.7	15.4
2005–2009	19.6	18.9	18.8	18.8	16.6	14.6	17.5	14.8	17.9	18.2
2010–2014	21.7	21.5	27.0	27.0	20.8	17.9	19.9	15.7	26.6	24.1
2015–2019	22.4	21.8	30.4	28.0	23.4	21.4	20.8	16.1	31.6	26.2

Because the unit of analysis was person-time, rather than person, the characteristics presented are weighted by a factor derived from the number of person-years of observation and number of events. For example, 45.3% of the person-years contributed by female candidates between 0 and 12 y were by people with congenital heart disease as the primary disease.

**TABLE 5.** Composition of the contrasted experience of female and male liver transplant candidates by candidate current age (unit of analysis is patient-years)

	0–12 y		13–24 y		25–44 y		45–59 y		≥60 y	
	Females	Males	Females	Males	Females	Males	Females	Males	Females	Males
Person-years of observation	4698	7606	24 123	30 595	226 005	300 826	359 999	547 504	346 203	514 300
Candidate race, %										
White	74.0	74.5	75.7	78.2	80.9	83.8	86.5	88.1	85.9	85.9
Black	18.3	17.4	17.1	15.1	13.3	8.7	8.7	6.0	7.4	6.4
Other	7.7	8.1	7.2	6.7	5.8	7.5	4.9	6.0	6.8	7.7
Primary disease										
Congenital biliary atresia	41.4	32.3	17.5	21.0	3.4	3.1	0.6	0.2	0.3	0.2
Alcoholic liver disease	0.0	0.0	0.1	0.2	10.9	17.6	17.1	24.1	13.3	26.6
Cancer	3.2	4.0	2.2	3.0	2.1	2.1	2.5	4.5	4.1	9.1
Metabolic liver disease	10.6	10.2	8.1	8.3	2.6	3.0	1.0	1.3	0.7	1.3
Fulminant liver failure	16.0	18.3	31.2	23.3	24.2	11.4	7.3	4.3	5.0	2.9
Autoimmune conditions	3.0	2.0	17.2	16.9	21.9	17.4	17.9	5.6	18.2	5.9
Hepatitis C	0.1	0.3	1.7	1.6	12.2	23.0	31.3	43.5	27.7	30.9
Others	25.8	32.9	22.1	25.6	22.6	22.5	22.2	16.6	30.6	23.1
Missing	8.5	8.3	12.5	11.5	8.9	6.3	3.3	1.8	3.7	2.7
Listing year, %										
1988–1994	9.6	9.2	4.4	3.2	5.0	5.5	2.5	1.7	1.3	0.9
1995–1999	16.1	15.8	11.1	9.6	14.6	16.9	10.2	9.7	5.9	4.4
2000–2004	20.9	20.8	17.7	16.7	19.3	20.6	21.0	21.3	12.9	10.1
2005–2009	19.8	21.0	21.7	22.3	18.7	17.9	23.3	24.5	18.7	16.5
2010–2014	18.3	18.8	23.9	25.4	20.9	19.2	23.5	24.1	27.6	29.6
2015–2019	15.2	14.5	21.2	22.7	21.5	19.8	19.5	18.7	33.7	38.5

Because the unit of analysis was person-time, rather than person, the characteristics presented are weighted by a factor derived from the number of person-years of observation and number of events. For example, 41.4% of the person-years contributed by female candidates between 0 and 12 y were by people with congenital biliary atresia as the primary disease.

### Relative Excess Mortality Risk by Candidate Sex

The interaction between sex and era was significant for waitlisted kidney (interaction *P* < 0.001) and liver (interaction *P* = 0.002), but not heart (interaction *P* = 0.6) candidates. For kidney, while the interaction between sex and era was statistically significant, the era-specific RER were similar to RER unstratified by era. For heart, the interaction between sex and era was not statistically significant and the era-specific RER were very similar to the RER unstratified by era. Therefore, results for kidney and heart candidates are shown without stratification by era. Era-stratified analyses for each organ are shown in (**Table S1, SDC**, https://links.lww.com/TXD/A791). In contrast, for liver transplant candidates, the RER in era-stratified models were not consistent across eras, suggesting that there have been changes over time. The plot showing mortality rates for male and female liver transplant candidates by calendar year of waitlist activation (**Figure S1, SDC**, https://links.lww.com/TXD/A791) shows similar or slightly higher mortality in males than females until about 2011, after which mortality was higher in females. We therefore present the results of liver candidates stratified into 2 eras: 1988–2011 and 2012–2019.

#### Kidney

**FIGURE 1.**
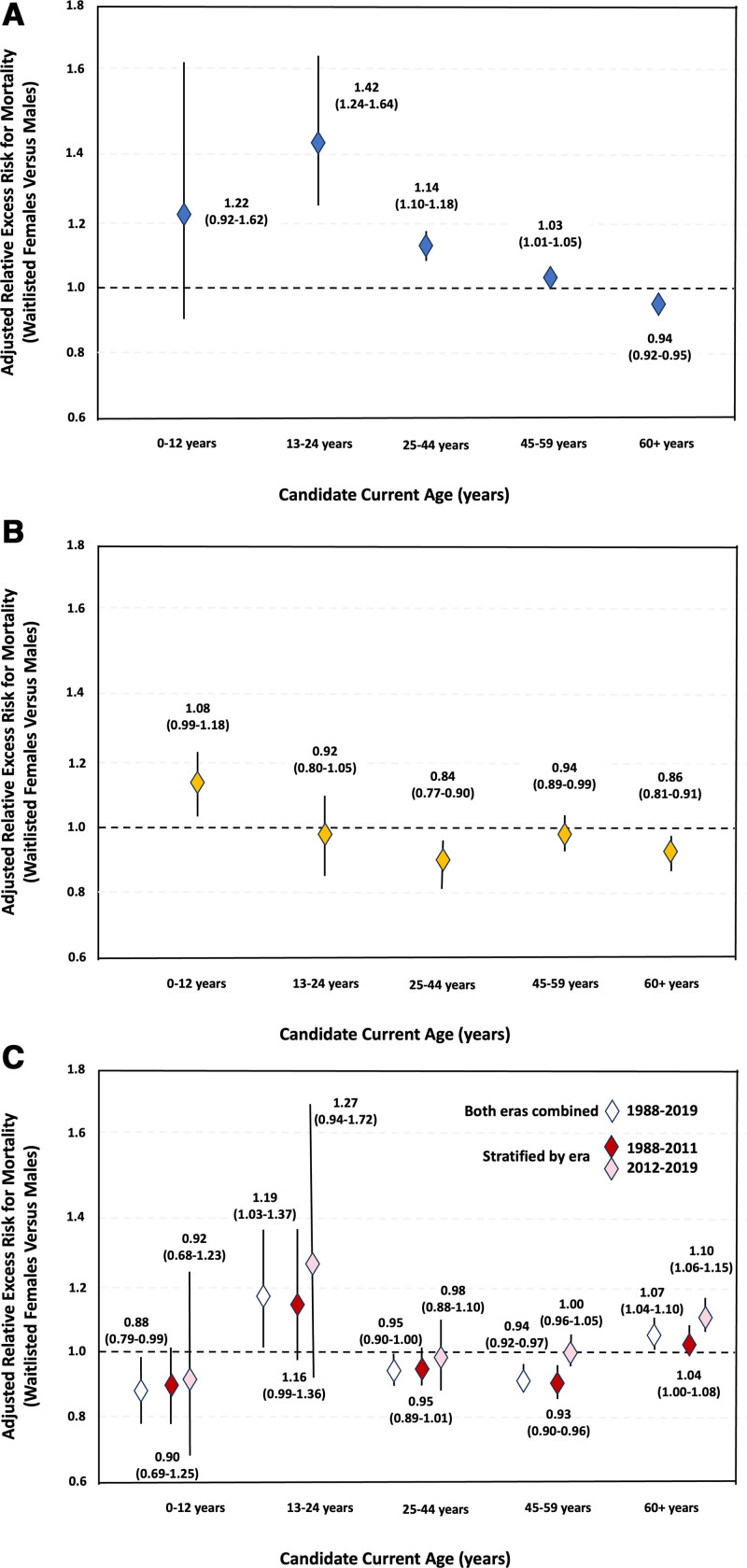
Adjusted relative excess risks (RER) of mortality in waitlisted female vs male (A) kidney, (B) heart, and (C) liver transplant candidates. The adjusted RERs of mortality in female vs male candidates in each age interval are shown, with 95% confidence intervals. Models were adjusted for candidate race (White, Black, other), primary disease (kidney: congenital anomalies of the kidneys and urinary tract [CAKUT], cystic kidney disease, glomerulonephritis, hypertension, diabetes, other; heart: congenital heart disease, coronary/ischemic heart disease, myocarditis/cardiomyopathy, other; liver: congenital biliary atresia, alcoholic liver disease, cancer, metabolic liver disease, fulminant liver failure, autoimmune conditions, hepatitis C, other), and the year listed for transplant. RER are analogous to hazard ratios and interpreted as excess risk of mortality associated with waitlisting for transplant in female candidates relative to that in males. Liver results are stratified by era (1988–2011, 2012–2019), but era-unstratified results are also shown as era-specific results were very similar among those <45 y old but limited in power.

As shown in Figure 1A, compared with waitlisted male kidney transplant candidates <60 y, female candidates of the same age experienced higher excess mortality, though the difference was not statistically significant among those <13 y (RER, 1.22; 95% CI, 0.92-1.62 for 0–12 y; RER, 1.42; 95% CI, 1.24-1.64 for 13–24 y; RER, 1.14; 95% CI, 1.10-1.18 for 25–44 y; and RER, 1.03; 95% CI, 1.01-1.05 for 45–60 y). In contrast, among candidates ≥60 y, females had lower excess mortality than males (RER, 0.94; 95% CI, 0.92-0.95). Results of unadjusted analyses, which were similar to adjusted results, are shown in (**Figure S2a, SDC**, https://links.lww.com/TXD/A791).

#### Heart

As shown in Figure 1B, among waitlisted heart transplant candidates ≥13 y old, females showed lower excess mortality than males (RER, 0.92; 95% CI, 0.80-1.05 for 13–24 y; RER, 0.84; 95% CI, 0.77-0.90 for 25–44 y; RER, 0.94; 95% CI, 0.89-0.99 for 45–60 y; RER, 0.86; 95% CI, 0.81-0.91 for ≥60 y); differences were only statistically significant among those ≥25 y. Among those under 13 y, females had slightly higher excess mortality than males (RER, 1.08; 95% CI, 0.99-1.18), though the difference was not statistically significant. Unadjusted analyses (**Figure S2b, SDC**, https://links.lww.com/TXD/A791) showed similar findings.

#### Liver

As shown in Figure 1C, among those <45 y, RER for the 2 eras (1988–2011 and 2012–2019) were similar. Among those 0–12 y (1988–2011: RER, 0.90; 95% CI, 0.79-1.02; 2012–2019: RER, 0.92; 95% CI, 0.68-1.23) and 25–44 y (1988–2011: RER, 0.95; 95% CI, 0.89-1.01 and 2012–2019: RER, 0.98; 95% CI, 0.88-1.10), females showed lower excess mortality than males in both eras, while among those 13–24 y (1988–2011: RER, 1.16; 95% CI, 0.99-1.36 and 2012–2019: RER, 1.27; 95% CI, 0.94-1.72), females showed higher excess mortality than males in both eras, though era-specific differences were not statistically significant for any of these age intervals. Among those 45–59 y, females waitlisted 1988–2011 had significantly lower excess mortality than males (RER, 0.93; 95% CI, 0.90-0.96), but there were no differences by sex among those waitlisted 2012–2019 (RER, 1.00; 95% CI, 0.90-0.96). There was slightly higher excess mortality in females than males ≥60 y waitlisted 1988–2011 (RER, 1.04; 95% CI, 1.00-1.08), but the difference was not statistically significant. Conversely, in the most recent era, excess mortality was significantly higher in females than males ≥60 y (RER, 1.10; 95% CI, 1.06-1.15). Unadjusted analyses are shown in (**Figure S2c, SDC**, https://links.lww.com/TXD/A791).

**FIGURE 2.**
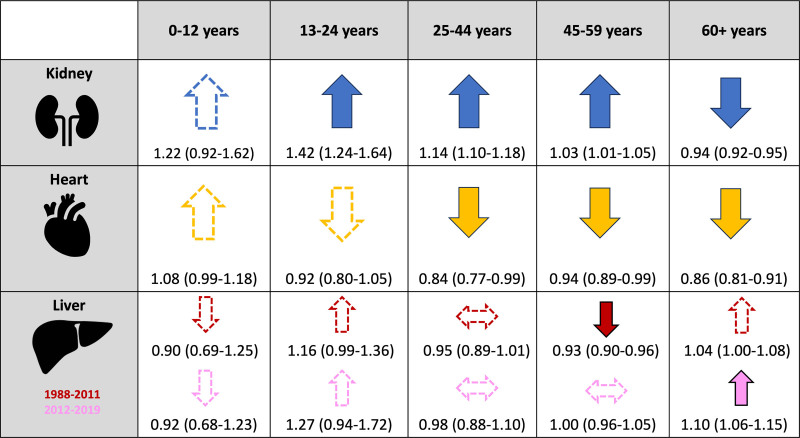
Summary of adjusted relative excess risks of mortality in waitlisted female vs male kidney, heart, and liver transplant candidates. Solid arrows indicate statistically significant differences; outlined arrows indicate the direction of the association where differences were not statistically significant.

Figure 2 summarizes differences in excess mortality risk between female and male candidates waitlisted for each organ type.

### Crude Excess and Absolute Mortality Rates

**FIGURE 3.**
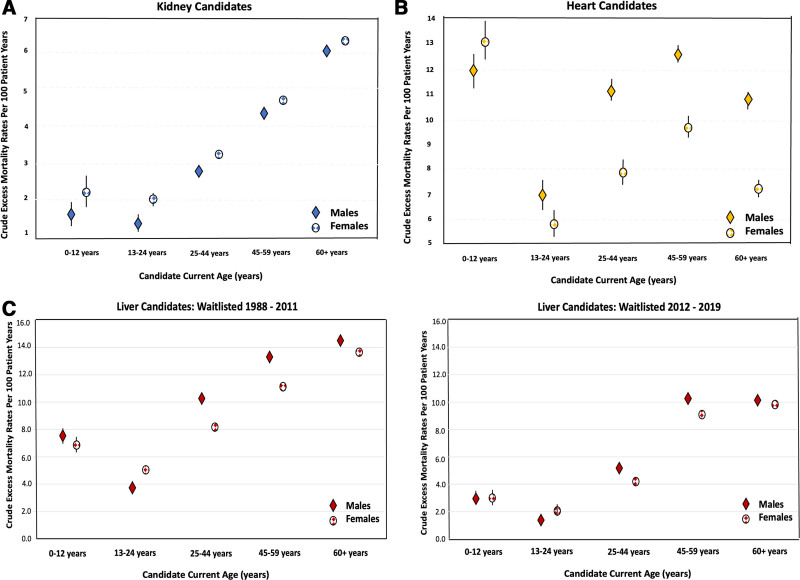
Crude excess mortality rates in female and male (A) kidney, (B) heart, and (C) liver transplant candidates. Crude excess mortality rates per 100 person-years of observation, with 95% confidence intervals, are shown within each age interval. Comparisons of crude excess mortality rates may be misleading as these comparisons do not take time since waitlisting into consideration. Rates for males are shown with solid diamonds and for females with open circles.

Crude excess mortality rates by organ type are shown in Figure 3; exact rates with 95% CIs are available in (**Table S2, SDC**, https://links.lww.com/TXD/A791). Absolute mortality rates are also shown in (**Table S2, SDC**, https://links.lww.com/TXD/A791). Even in the candidate age categories where there were statistically significant differences in excess mortality rates, the absolute differences by candidate sex were fairly small; the greatest absolute differences by sex were observed for heart transplant candidates aged 25–44 y (7.9 per 100 patient-years, 95% CI, 7.4-8.4 for females and 11.2, 95% CI, 10.8-11.7 for males; absolute difference in excess mortality 3.3 per 100 person-years) and ≥60 y (7.2 per 100 patient-years, 95% CI, 6.8-7.7 for females and 10.8, 95% CI, 10.5-11.1 for males; absolute difference in excess mortality 3.6 per 100 person-years). Comparisons of crude excess mortality rates by sex may be misleading (compared with models) as they do not take time since waitlisting into account.

## DISCUSSION

Numerous factors may contribute to sex differences in excess mortality among patients waitlisted for organ transplant. These include sex disparities in referral for transplant evaluation, completion of evaluation, activation on the waitlist, and transplantation,^23-25^ as well as differences in the care provided to males and females with organ failure.^24,26^ The results of this study must be interpreted in the context of what is already known about sex disparities among transplant candidates. A 2023 conference report highlighted that, for all organs, women are less likely than men to be referred for transplant evaluation, to complete evaluation, and to be activated on the waitlist.^24,25,27-30^ Access to transplant is particularly limited for women at older ages,^23,25,28-31^ even among women with comparatively low comorbidity burden.^28,32,33^ A recent Canadian study demonstrated that even in the absence of an absolute contraindication to transplant, women with ESKD ≥60 y have 68% lower odds of referral for kidney transplant evaluation than age-matched men.^25^ Disparities in access to the waitlist are likely to result in selection bias whereby waitlisted women are systematically healthier than waitlisted men.

We found higher excess mortality among female than male candidates <60 y waitlisted for kidney transplant, but lower excess mortality in female than male candidates ≥60 y. Previous studies of patients with ESKD showed higher excess mortality in females than males, with the largest RER and standardized mortality ratios in the youngest patients.^26,34-37^ There is evidence that girls and women with ESKD may be systematically under-dialyzed compared with boys and men because of the lower volume of distribution of urea per unit body surface area in females, potentially contributing to higher mortality in females.^26^ Given that patients waitlisted for kidney transplant have ESKD—with most on dialysis—it was not surprising to find higher excess mortality among females than males of most ages waitlisted for kidney transplant. However, it is important to recognize that these previous studies were not restricted to patients activated on the transplant waitlist—a select healthier population. Bias in the selection of older women for activation on the waitlist wherein, systematically, healthier older women than men are waitlisted most likely explains the lower excess mortality in female than male candidates ≥60 y. The observed sex differences in excess mortality appeared relatively stable over the entire observation interval, though there was some widening of the gaps in the most recent era.

We showed significantly lower excess mortality in female than male heart transplant candidates ≥25 y old. This observation is likely driven by the known lower mortality rates in women listed as United Network for Organ Sharing (UNOS) status 2^38^; status 2 candidates comprise the majority of patients waitlisted for heart transplant. The reasons for lower mortality in status 2 women than men are incompletely understood but likely reflect systematic bias in access to the waitlist, wherein healthier women than men are waitlisted. Although women comprise 54% of heart failure deaths, they make up only 21% of left ventricular assist device recipients and only 25% of heart transplant candidates^39,40^ and are less likely to be referred for heart transplant.^31,39^ An observational cohort study demonstrated that compared with male heart transplant recipients, at the time of transplant, females were younger, less likely to have diabetes, dyslipidemia, hypertension, or ischemic cardiomyopathy.^41^ Interestingly, women with UNOS status 1A heart failure (the most critically ill patients) exhibit greater mortality than males with the same status.^38^ Female heart transplant candidates often present more acutely for transplant than males, with greater requirements for pretransplant inotropy and mechanical ventilation at listing.^13,38^ It has been suggested that women may be less likely than men to be successfully bridged to transplant with ventricular assist devices or artificial hearts and may be more likely to be inactivated on the waitlist.^38^

The observed sex differences in excess mortality among liver transplant candidates are more complicated; sex differences differed by age and were not always consistent across calendar time. It is important to situate our findings in the context of the current understanding of sex difference in access to liver transplantation, the impact of liver allocation policies on sex differences in mortality, as well as how changes to allocation policies over time may have influenced changes in mortality risks.

There is a general consensus that women are less likely than men to be referred, evaluated, and activated on the liver transplant waitlist.^24,30^ Bias in referral and waitlisting is most marked for older transplant candidates.^25^ Whether this bias extends to adolescents and young adults is unknown; as in kidney transplant,^25^ bias may be less serious or absent among younger liver candidates. Most previous studies also showed higher mortality in females than males with advanced liver disease.^14,42,43^ It is also well known that the model for end-stage liver disease (MELD) score-based liver allocation disadvantages women, contributing to higher mortality among female than male candidates and recipients.^14,44^ It is important to recognize that no previous studies comparing mortality by sex were stratified by candidate or recipient age; higher mortality rates observed for women versus men were likely driven by the oldest women, who make up the largest proportion of all candidates and recipients. Furthermore, we acknowledge that MELD scores are not relevant for candidates <12 y.

MELD-based liver allocation was adopted in the United States in 2002. In an effort to reduce regional disparities and improve transplant access for the sickest patients, in 2010, regional sharing for status 1 candidates was implemented; status 1 patients (predicted to die within 7 d without transplant) got priority over all others in the UNOS region. In 2012, policies known as Regional Share 35 and National Share 15 gave additional priority to patients with MELD scores >35 and >15, respectively.^45^ We noted a change in the patterns of sex disparity after 2012, suggesting that these well-intentioned modifications to allocation may have unwittingly resulted in greater sex disparities in mortality. Given that women are known to be sicker than men with the same MELD score,^44^ these policy changes may have directed livers away from women to men with higher MELD scores based only on their sex.

We found lower excess mortality in female than male liver candidates <13 y, which was similar in both eras, though not statistically significant when stratified by era. The reasons for this difference are not clear and deserve further investigation; it seems unlikely that selection bias in waitlisting would play a role in these observations in the pediatric population. Female liver candidates 13–24 y showed higher excess mortality than male candidates of the same age; the magnitude of the difference was similar across eras. If there was no selection of healthier girls and young women for waitlisting, the higher excess mortality among waitlisted candidates in this age group may reflect the fact that females have lower MELD scores than men with a similar level of illness and therefore die without receiving an offer more often.

There were no clear differences in excess mortality by sex among candidates 25–44 y. In this age interval, perhaps a bias toward healthier women being selected for waitlisting is countered by a higher mortality risk because of inequities introduced by the MELD score, resulting in no difference in excess mortality overall. Otherwise stated, despite the fact that waitlisted women in this age group are healthier overall than waitlisted men, they have similar excess mortality rates. Among candidates 45–59 y, females showed significantly lower excess mortality than males in the more remote era, but no differences in the most recent era. Among those ≥60 y, females showed slightly, but not significantly, higher excess mortality than males in the more remote era but significantly higher excess mortality than males after 2012. A potential explanation for these observations is similar to that proposed for those 25–44 y wherein selection of healthier women for waitlisting is countered, to a greater or lesser degree, by the higher mortality risk associated with the inequities introduced by MELD scores. The differences by era may be because of a greater impact of MELD-based disparities after the introduction of Regional Share 35 and National Share 15 in 2012, which likely disproportionately advantaged men and disadvantaged women. Although these hypotheses for the reasons underlying the observed sex differences in excess mortality are as yet unproven, our observations highlight the importance of assessing the impact of organ allocation policy changes separately for males and females.

Although this study has many strengths, limitations must be considered. As with all retrospective analyses, this study was at risk for miscoding and misclassification of data; however, we would anticipate any errors in data to be nondifferentially distributed and unlikely to bias results. We did not have reliable data regarding the cause of death and therefore could not examine if there were differences in excess cause-specific mortality by sex, as shown in the earlier study of sex differences in mortality risk in Australian and New Zealand patients with ESKD.^9^ In our previous study assessing sex differences in graft loss and mortality risk after transplant, we used individual patient data meta-analyses to combine data from 3 transplant registries (the SRTR, the Collaborative Transplant Study, and the Australia and New Zealand Dialysis and Transplant Registry).^6-8,46^ Unfortunately, waitlist data were not available in the Collaborative Transplant Study, and Australia and New Zealand Dialysis and Transplant Registry only has waitlisting data for kidney and numbers are relatively small compared with SRTR. Therefore, our study was restricted to the United States (SRTR), limiting generalizability to other countries including those with socialized healthcare.

## CONCLUSIONS

The patterns of sex differences in excess mortality among kidney, heart, and liver transplant candidates of different ages, interpreted in the context of previous study showing restricted access to the waitlist for women compared with men and higher excess mortality rates among females than males with ESKD, heart failure, and liver failure, suggest that bias in the selection of adult patients for activation on the waitlist may be at play, wherein systemically healthier women than men make it through to the transplant waitlist. It appeared that such selection bias was most severe in the oldest patients. We observed no evidence that similar selection bias occurred in the pediatric population. In liver transplantation, higher excess mortality in male than female pediatric patients was observed; this deserves more in-depth examination to understand the reasons. We noted changes over time in the patterns of sex differences in excess mortality among liver transplant candidates and hypothesize that changes to allocation policy around 2012 may have amplified sex disparities in mortality risk related to MELD-based allocation.^24^ These findings highlight the importance of studies assessing the sex-specific impact of organ allocation policy changes. Ultimately, this observational study can only reveal patterns of sex differences; it is not possible to determine the mechanisms underlying the differences. The hypothesized mechanisms need to be confirmed in future studies.

## Supplementary Material
