# Supplementary material for: Sex Differences in Excess Mortality Among Waitlisted Kidney, Heart, and Liver Transplant Candidates
Source: Transplant Direct. 2025 Aug 22;11(9):e1856. doi: 10.1097/TXD.0000000000001856 (PMC12377303; doi:10.1097/TXD.0000000000001856)
Supplement: Supplementary file 1 [file txd-11-e1856-s001.pdf]

List of Supplementary Materials:

Table S1: Relative Excess Risk for Females versus Males Waitlisted for Kidney, Liver, and Heart Transplant by Era (1988-1998; 1999-2008; 2009-2019).

Table S2: Crude excess mortality rates and absolute mortality rates in Female and Male Kidney, Heart , and Liver Transplant Candidates within each age interval

The total number of person-years of observation, total observed number of deaths, absolute mortality rates, expected number of deaths, excess deaths, and crude excess mortality rates, with 95% confidence intervals, are shown for each candidate sex and age interval.

Figure S1 : Plot of mortality rate by calendar year for male and female liver transplant candidates.

Absolute mortality rates per 100 person-years of observation by calendar year of listing are shown for male (orange) and blue (female) liver transplant candidates.

Figure S2: Unadjusted Relative Excess Risks of Mortality in Waitlisted Female versus Male (a) Kidney, (b) Heart, and (c) Liver Transplant Candidates (listed 1988-2011 and listed 2012-2019 separately). The unadjusted relative excess risks (RER) of mortality in female versus male candidates in each age interval are shown, with 95% confidence intervals.

Table S1: Relative Excess Risk for Females versus Males Waitlisted for Kidney, Liver, and Heart Transplant by Era (1988-1998; 1999-2008; 2009-2019).

	1988-1998	1999-2008	2009-2019
**Kidney**			
**0-12 years**	1.24 (0.62-2.48)	1.17 (0.75-1.81)	1.47 (0.92-2.34)
**13-24 years**	**1.39 (1.05-1.82)**	**1.39 (1.13-1.17)**	**1.43 (1.09-1.86)**
**25-44 years**	**1.13 (1.07-1.20)**	**1.16 (1.10-1.22)**	**1.13 (1.06-1.21)**
**45-59 years**	**1.07 (1.03-1.11)**	1.02 (0.99-1.05)	1.00 (0.97-1.04)
**60+ years**	0.99 (0.96-1.03)	**0.91 (0.89-0.94)**	**0.90 (0.88-0.92)**
**Liver**			
**0-12 years**	0.83 (0.67-1.03)	0.90 (0.77-1.05)	1.02 (0.81-1.29)
**13-24 years**	0.98 (0.74-1.32)	1.20 (0.98-1.48)	**1.28 (1.01-1.63)**
**25-44 years**	0.96 (0.86-1.08)	0.94 (0.87-1.01)	0.94 (0.86-1.03)
**45-59 years**	0.99 (0.92-1.07)	**0.90 (0.87-0.93)**	**0.95 (0.91-0.98)**
**60+ years**	0.91 (0.82-1.01)	1.01 (0.97-1.06)	1.03 (1.00-1.06)
**Heart**			
**0-12 years**	1.15 (1.00-1.33)	1.09 (0.93-1.27)	1.05 (0.90-1.22)
**13-24 years**	0.94 (0.75-1.19)	0.90 (0.71-1.13)	0.94 (0.71-1.25)
**25-44 years**	**0.87 (0.77-0.98)**	0.88 (0.77-1.01)	**0.78 (0.67-0.89)**
**45-59 years**	**0.81 (0.74-0.89)**	1.00 (0.91-1.09)	1.01 (0.92-1.11)
**60+ years**	**0.73 (0.63-0.84)**	**0.88 (0.79-0.98)**	**0.86 (0.79-0.94)**

**Interaction between sex and era (1988-1998; 1999-2008; 2009-2019): Kidney (p-value 0.0005); Liver (p-value 0.0023); Heart (p-value 0.6308).*

Table S2: Crude absolute and excess mortality rates in Female and Male Kidney, Heart, and Liver Transplant Candidates within each age interval

	0-12 years		13-24 years		25-44 years		45-59 years		≥60 years	
	Females	Males	Females	Males	Females	Males	Females	Males	Females	Males
**Kidney Candidates**										
**Person-years of observation**	4698	7606	24123	30595	226005	300826	359999	547504	346203	514300
**Deaths**	105	127	498	469	7779	9172	18869	28315	27101	43451
**Absolute Mortality Rate/100 pt years**	2.2	1.7	2.1	1.5	3.4	3.0	5.2	5.2	7.8	8.4
**Expected Deaths***	1.2	3.0	10.8	40.4	306.8	780.0	1868.2	4703.5	5377.6	12229.7
**Excess deaths**	103.8	124.0	487.2	428.6	7472.2	8392.0	17000.8	23611.5	21723.4	31221.3
**Crude excess mortality rate per 100 yrs (95% CI)**	**2.2** 1.8-2.7	**1.6** 1.4-1.9	**2.0** 1.8-2.1	**1.4** 1.3-1.5	**3.3** 3.2-3.4	**2.8** 2.7-2.9	**4.7** 4.6-4.8	**4.3** 4.3-4.4	**6.3** 6.2-6.4	**6.1** 6.0-6.1
**Heart Candidates**										
**Person-years of observation**	7214	9320	5127	7641	11748	20853	17372	53608	14557	51183
**Deaths**	947	1123	302	543	938	2394	1788	7230	1273	6630
**Absolute Mortality Rate/100 pt years**	13.1	12.0	5.9	7.1	8.0	11.5	10.3	13.5	8.7	13.0
**Expected Deaths**	6.0	9.2	1.8	7.3	14.9	51.3	89.7	457.3	218.3	1114.6
**Excess deaths**	941.0	1113.8	300.2	535.7	923.1	2342.7	1698.3	6772.7	1054.7	5515.4
**Crude excess mortality rate per 100 yrs (95% CI)**	**13.0** 12.3-13.8	**12.0** 11.3-12.6	**5.8** 5.3-6.5	**7.0** 6.5-7.6	**7.9** 7.4-8.4	**11.2** 10.8-11.7	**9.8** 9.3-10.2	**12.6** 12.4-12.9	**7.2** 6.8-7.7	**10.8** 10.5-11.1
**Liver Candidates**										
**Waitlisted 1988 - 2011**										
**Person-years of observation**	8914	9626	9182	8041	28101	30043	65221	102016	41563	44712
**Deaths**	619	738	470	314	2329	3168	7554	14365	6242	7362
**Absolute Mortality Rate/100 pt years**	6.9	7.7	5.1	3.9	8.3	10.5	11.6	14.1	15.0	16.5
**Expected Deaths**	9.8	11.8	3.5	7.9	32.4	66.3	295.6	737.9	550.8	834.6
**Excess deaths**	609.2	726.2	466.5	306.1	2296.6	3101.7	7258.4	13627.1	5691.2	6527.4
**Crude excess mortality rate per 100 yrs (95% CI)**	**6.8** 6.3−7.4	**7.5** 7.0−8.1	**5.1** 4.7−5.5	**3.8** 3.4−4.2	**8.2** 7.9−8.5	**10.3** 10.0−10.7	**11.1** 10.9−11.4	**13.4** 13.2−13.6	**13.7** 13.4−14.0	**14.6** 14.3−14.9
**Waitlisted 2012 - 2019**										
**Person-years of observation**	3100	3243	5101	4967	14698	13919	32693	49702	44098	62100
**Deaths**	96	98	109	75	632	750	3123	5511	4940	7483
**Absolute Mortality Rate/100 pt years**	**3.1**	**3.0**	**2.1**	**1.5**	**4.3**	**5.4**	**9.6**	**11.1**	**11.2**	**12.1**
**Expected Deaths**	2.0	2.1	1.8	4.4	16.3	28.3	155.8	388.4	606.6	1172.8
**Excess deaths**	94.0	95.9	107.2	70.6	615.7	721.7	2967.2	5122.6	4333.4	6310.2
**Crude excess mortality rate per 100 yrs (95% CI)**	**3.0** 2.5−3.7	**3.0** 2.4−3.6	**2.1** 1.7−2.5	**1.4** 1.1−1.8	**4.2** 3.9−4.5	**5.2** 4.8−5.6	**9.1** 8.8−9.4	**10.3** 10.0−10.6	**9.8** 9.6−10.1	**10.2** 9.9−10.4

The total number of person-years of observation, total observed number of deaths, expected deaths, excess deaths, and crude excess mortality rates, with 95% confidence intervals, are shown for each candidate sex and age interval.

*Expected numbers of deaths are based on the age-, sex-, race-, and calendar year-specific mortality rates in the general population and the distributions of these in the waitlisted candidates.

Figure S1 : Plot of mortality rate by calendar year for male and female liver transplant candidates

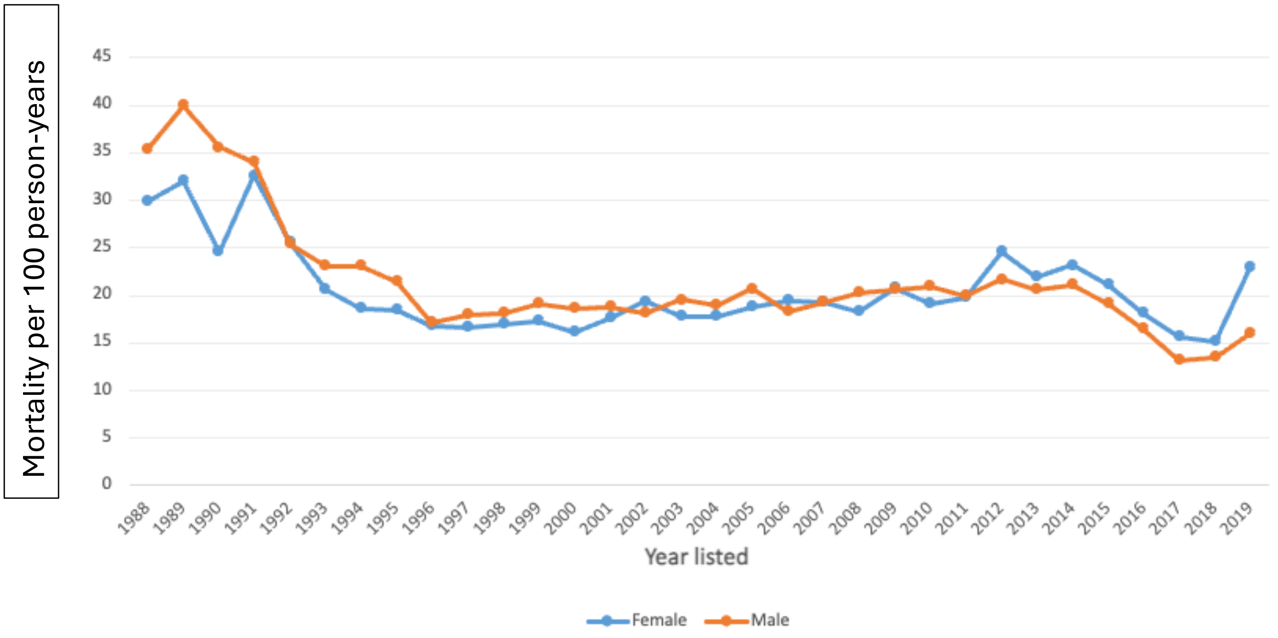

Figure S2a: Unadjusted Relative Excess Risks of Mortality in Waitlisted Female versus Male Kidney Transplant Candidates.

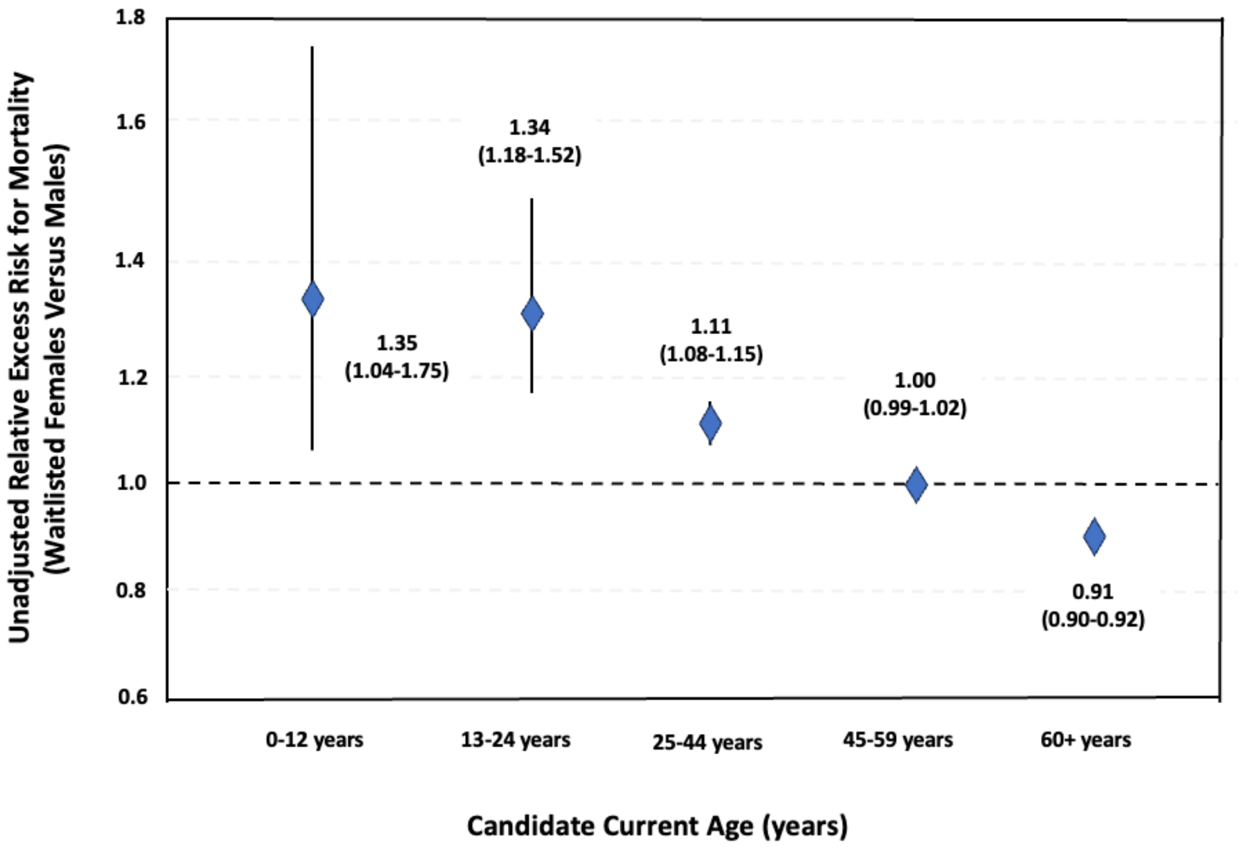

The unadjusted relative excess risks (RER) of mortality in female versus male kidney transplant candidates in each age interval are shown, with 95% confidence intervals.

Figure S2b: Unadjusted Relative Excess Risks of Mortality in Waitlisted Female versus Male Heart Transplant Candidates.

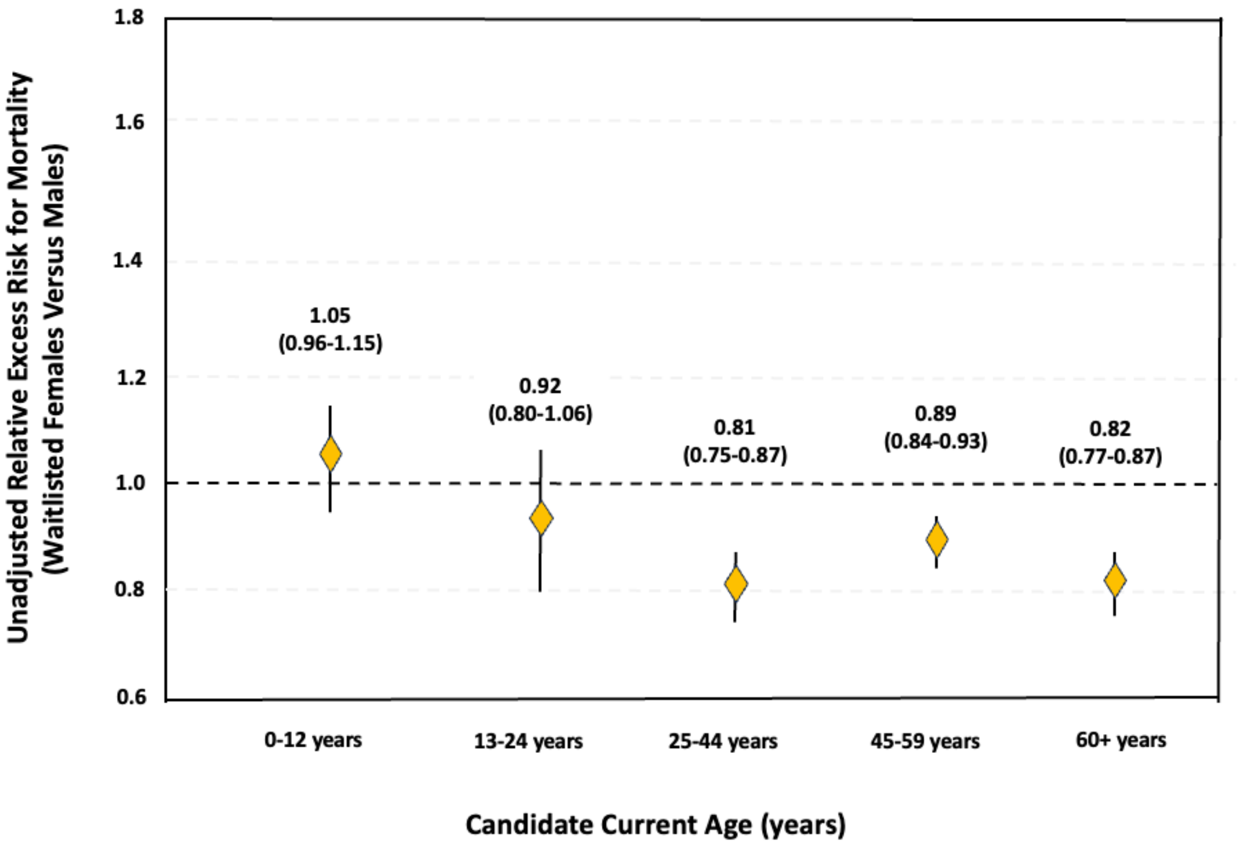

The unadjusted relative excess risks (RER) of mortality in female versus male heart transplant candidates in each age interval are shown, with 95% confidence intervals.

Figure S2c: Unadjusted Relative Excess Risks of Mortality in Waitlisted Female versus Male Liver Transplant Candidates.

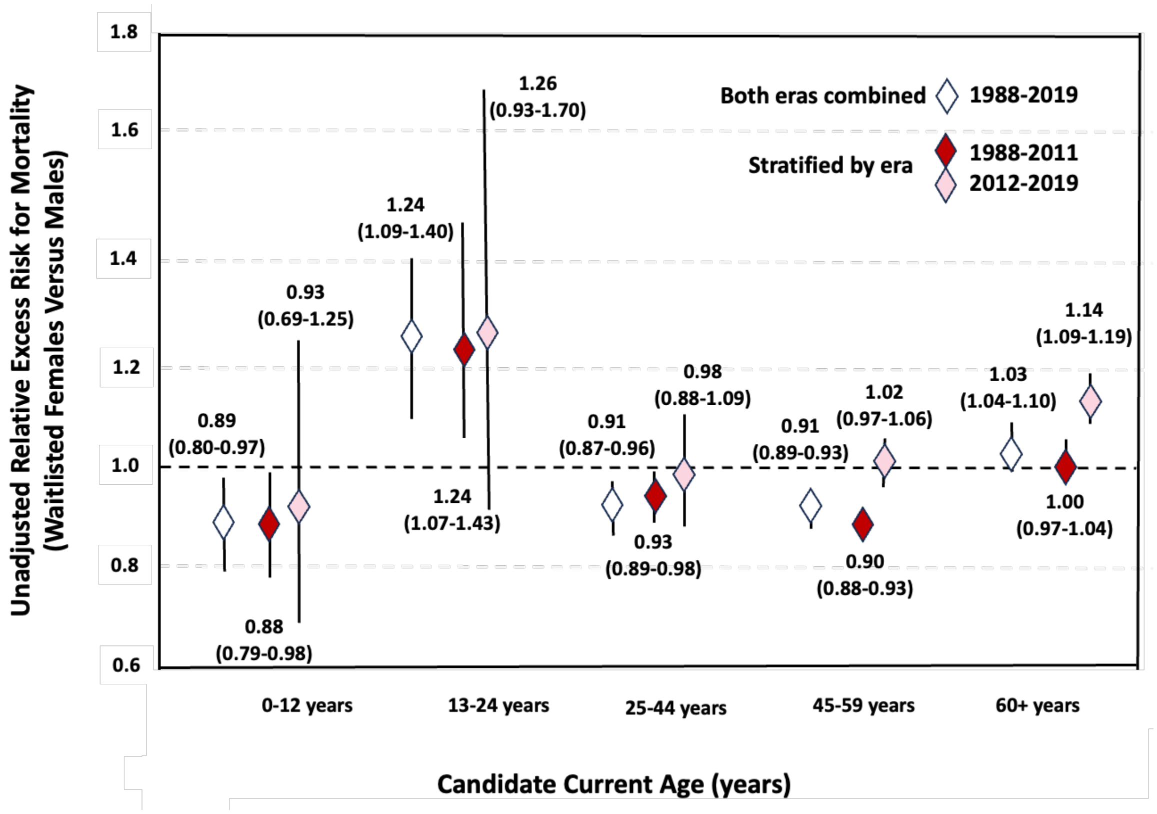

The unadjusted relative excess risks (RER) of mortality in female versus male liver transplant candidates in each age interval are shown, with 95% confidence intervals.
